# Greenhouse Gas Emissions from Cotton Field under Different Irrigation Methods and Fertilization Regimes in Arid Northwestern China

**DOI:** 10.1155/2014/407832

**Published:** 2014-07-16

**Authors:** Jie Wu, Wei Guo, Jinfei Feng, Lanhai Li, Haishui Yang, Xiaohua Wang, Xinmin Bian

**Affiliations:** ^1^College of Agriculture, Nanjing Agricultural University, Nanjing 210095, China; ^2^State Key Laboratory of Desert and Oasis Ecology, Xinjiang Institute of Ecology and Geography, Chinese Academy of Sciences, Xinjiang 830011, China

## Abstract

Drip irrigation is broadly extended in order to save water in the arid cotton production region of China. Biochar is thought to be a useful soil amendment to reduce greenhouse gas (GHG) emissions. Here, a field study was conducted to compare the emissions of nitrous oxide (N_2_O) and methane (CH_4_) under different irrigation methods (drip irrigation (D) and furrow irrigation (F)) and fertilization regimes (conventional fertilization (C) and conventional fertilization + biochar (B)) during the cotton growth season. The accumulated N_2_O emissions were significantly lower with FB, DC, and DB than with FC by 28.8%, 36.1%, and 37.6%, while accumulated CH_4_ uptake was 264.5%, 226.7%, and 154.2% higher with DC, DB, and FC than that with FB, respectively. Irrigation methods showed a significant effect on total global warming potential (GWP) and yield-scaled GWP (*P* < 0.01). DC and DB showed higher cotton yield, water use efficiency (WUE), and lower yield-scaled GWP, as compared with FC and FB. This suggests that in northwestern China mulched-drip irrigation should be a better approach to increase cotton yield with depressed GHG. In addition, biochar addition increased CH_4_ emissions while it decreased N_2_O emissions.

## 1. Introduction

Crop cultivation stimulates greenhouse gas (GHG) emissions from soil to the atmosphere from agricultural practices such as irrigation and fertilization, which in turn influences the biogeochemical process of carbon and nitrogen (N) in the soil. The emissions of GHG from crop land have been estimated to account for 13.5% of the anthropogenic emissions worldwide [1]. How to reduce GHG emissions from agricultural practices without yield loss is an urgent task for crop production. Improving the cropping practices is a recommended strategy to mitigate greenhouse gas emissions from agricultural soil [1]. However, this strategy is highly dependent on the crops, since the cropping practices varied with crop species [2]. Cotton is one of the major cash crops delivering natural fibers to textile industries around the world. Globally, the harvested area of seed cotton is 32 million ha in 2010 [3]. Considerable field experiments have documented large amount of N_2_O emitted from cotton field due to high N fertilizer input and immoderate irrigation [4–6].

Soil moisture is one of the key factors affecting GHG production in agricultural soil. An optimal irrigation can reduce GHG emissions by regulating the N and carbon turnover process in soil via manipulating soil moisture. Most of the cotton production is situated in the semiarid or arid areas, where water-saving irrigation is a key issue for the cotton cultivation. Drip irrigation is one of the water-saving irrigation approaches broadly extended in semiarid or arid regions, since it can reduce surface evaporation, surface runoff, and deep percolation [7]. Water and mineral N fertilizer are directly supplied to the crop root zone through drip irrigation system to adapt to the crop requirements, hence improving the water and N use efficiency. Therefore, drip irrigation may have a large influence on the nitrogen and carbon turnover in soil and reduce the N fertilizer-induced N_2_O or carbon-related greenhouse gas (e.g., CH_4_) production, in relation to conventional furrow irrigation. For instance, several studies showed that drip irrigation significantly decreased the N_2_O emission from tomato and melon field, as compared with furrow irrigation [8–10]. However, the N fertilizer application rate is much higher in cotton cultivation than that in the aforementioned crops. The N fertilizer application rate is approximately 300 kg N ha^−1^ in cotton production area of China, which is nearly two times higher than that in previous studies (120–175 kg N ha^−1^) [8, 9]. Thus, it is still unknown what the impact of drip irrigation would be on N_2_O emission under high N fertilizer application conditions.

Biochar is the byproduct of biomass pyrolysis, one of the technologies used to produce bioenergy. It has been suggested that biochar can be a useful soil amendment to improve soil physiochemical properties and crop yield, as well as to increase soil carbon storage and reduce GHG emissions [11–13]. Biochar addition could mitigate or inhibit N_2_O emission in most studies for increased adsorption of NH_4_^+^ or changes in pH that alter the N_2_O-to-N_2_ ratio during denitrification [14–16]. However, the effects of biochar on CH_4_ emissions have yet been inconsistent. Previous study showed that biochar addition to the upland soil increased CH_4_ emissions by 37% [17]. On the other hand, CH_4_ uptake increased in some studies after biochar additions [18, 19]. The results for the observed changes in CH_4_ emissions may contradictorily depend on soil water content, soil type, and biochar type. Till now, few data are available to support these conclusions on the field scale especially for uplands.

Drip irrigation with plastic film mulching is widely recommended as a replacement of the traditional furrow irrigation, because seasonal shortage of irrigation water and low temperature have become critical factors limiting the productivity of cotton crop in this area. However, only a few studies investigated the characteristics of CO_2_, N_2_O, and CH_4_ emissions from cotton field under drip irrigation in China [20–22]. To our knowledge, there are no published field studies on the effect of biochar addition on GHG emissions from cotton field. Thus, the objectives of this study are to (a) investigate the characteristics of N_2_O and CH_4_ emissions from cotton field under different irrigation methods and fertilization regimes and (b) compare the integrated effects of different irrigation methods and fertilization regimes on the GHG emissions.

## 2. Materials and Methods

### 2.1. The Study Site

A field experiment was carried out at the experimental farm of Shihezi University in Xinjiang Province (45°19′ N, 116°34′ E, 433–437 m in elevation), which locates in the primary cotton production region of China. This region has a dry continental climate with mean annual temperature of 8°C and precipitation of 150 mm, most of which occurs from June to September. The main crops in this area are cotton, wheat, and maize. The soil in the experiment site is heavy loam, and the previous crop is cotton. Some chemical properties for the topsoil sampled at 0–15 cm depth were as follows: soil organic matter, 13.1 g*·*kg^−1^; total soil nitrogen, 0.9 g*·*kg^−1^; available soil phosphorus, 66.3 mg*·*kg^−1^; available soil potassium, 169.8 mg*·*kg^−1^.

### 2.2. Treatments and Field Work

**Figure 1:**
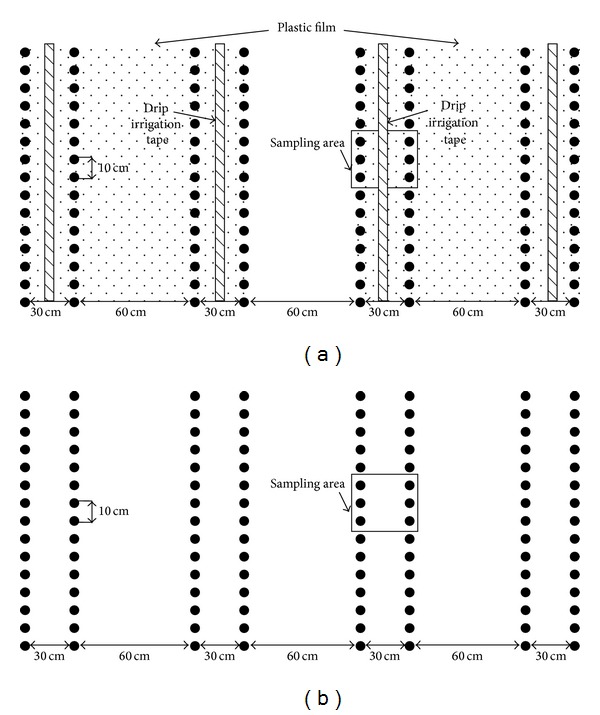
Experimental layout in the cotton field for drip irrigation treatments (DC, DB) (a) and furrow irrigation treatments (FC, FB) (b).

The field experiment comprised two factors during the cotton growing seasons of 2011, including different irrigation methods (furrow irrigation and drip irrigation) and fertilization regimes (conventional fertilization and conventional fertilization + biochar). The four treatments included (1) FC, furrow irrigation (mulch-free) with conventional fertilization; (2) DC, drip irrigation (plastic film mulching) with conventional fertilization; (3) FB, furrow irrigation (mulch-free) with conventional fertilization + biochar; (4) DB, drip irrigation (plastic film mulching) with conventional fertilization + biochar. The experiment was a randomized block design with three replicates. The size of each experimental plot was 40 m^2^ (5 m × 8 m). As shown in Figure 1, the cotton was planted in narrow row spacing of 30 cm and wide row spacing of 60 cm, with plant spacing of 10 cm. For treatments of DC and DB, transplant plastic film in width of 120 cm covered four rows.

Seeds (Xinjiang cotton cv. number 36) were sown on April 27 and emerged on May 5. The fertilization and irritation were applied according to the local farming regime. The irrigation volumes were 4500 m^3 ^ha^−1^ and 6000 m^3 ^ha^−1^ for drip irrigation treatments (DC and DB) and furrow irrigation treatments (FC and FB), respectively. The biochar (Sanli New Energy, China) was applied as basal fertilizer at a rate of 7500 kg hm^−2^. Chemical fertilizer was applied at the same total rate of diammonium phosphate (300 kg*·*hm^−2^), urea (555 kg*·*hm^−2^), and potassium dihydrogen phosphate (90 kg*·*hm^−2^) for all treatments. Diammonium phosphate was applied as basal fertilizer. The percentages for dressing fertilizer were different for two irrigation methods: the topdressing was applied in three times from June 10 to July 10 for furrow irrigation treatments, while the topdressing was fertigated with the drip irrigation system in several times for the drip irrigation treatments. The detail of fertilization and irrigation for these four treatments was shown in Table 1.

### 2.3. Investigation of GHG Emissions

GHG fluxes from cotton field were measured using static chamber and gas chromatography method [23]. The size of chambers was 80 cm × 80 cm × 45 (90) cm (length × width × height); the height of chambers was adapted to cotton plant growth. The gas sampling was carried out between 9:00 and 11:00 hours. Gas samples were drawn from the chambers through a three-way stopcock using an airtight syringe with volume of 50 mL at 0, 10, 20, and 30 min after closure and immediately transferred into 50 mL vacuum glass container. The GHG fluxes from all plots were measured at 7-day interval. The gas samples were analyzed for the concentrations of N_2_O and CH_4_ using a gas chromatograph (Agilent 7890, Agilent Technologies, USA) equipped with an electron capture detector (ECD) and a flame ionization detector (FID). The rates of N_2_O and CH_4_ flux were calculated by the linear increase of the gas concentration at each sampling time (0, 10, 20, and 30 min); sample sets were rejected unless the correlation coefficient (*R* ^2^) for the linear regression is greater than 0.9 (0.7 for small flux rates). All flux rates were adjusted for air temperature, air pressure, and area and volume of the chamber [24]. Average GHG fluxes were calculated by triplicate plots. Seasonal accumulation amounts of GHG emissions were calculated by the emissions between every two adjacent intervals of the measurements.

### 2.4. Soil Temperature, Moisture, and Mineral N Content

Soil temperature and soil moisture were measured at four different points near the area covered by the chamber. Soil temperature was taken at 5 cm depth. Soil moisture was determined using a TDR (time domain reflectometer) [25].

Surface soil samples (0–20 cm) at the experiment plots close to the chamber covered area were collected for the analysis of soil mineral N (ammonium and nitrate) contents at the same day as the gas sampling during the cotton growth season. Fresh soil samples were extracted with 0.01 M CaCl_2_ in a 1 : 10 ratio of soil to extractant. The concentrations of ammonium and nitrate in the extract were analyzed using continuous flow analytical system [26]. Cotton yield was recorded at cotton harvest.

### 2.5. Statistical Analyses

Differences in seasonal N_2_O and CH_4_ emissions, soil temperature, soil N mineral contents, and cotton yield as affected by irrigation methods and fertilization regimes were examined by using a two-way analysis of variance (ANOVA). The statistical analysis was carried out using SPSS 20.0 (IBM SPSS Statistics, Chicago, IL, USA).

## 3. Results

### 3.1. Soil Characteristics, Cotton Yield, and Water Use Efficiency

**Figure 2:**
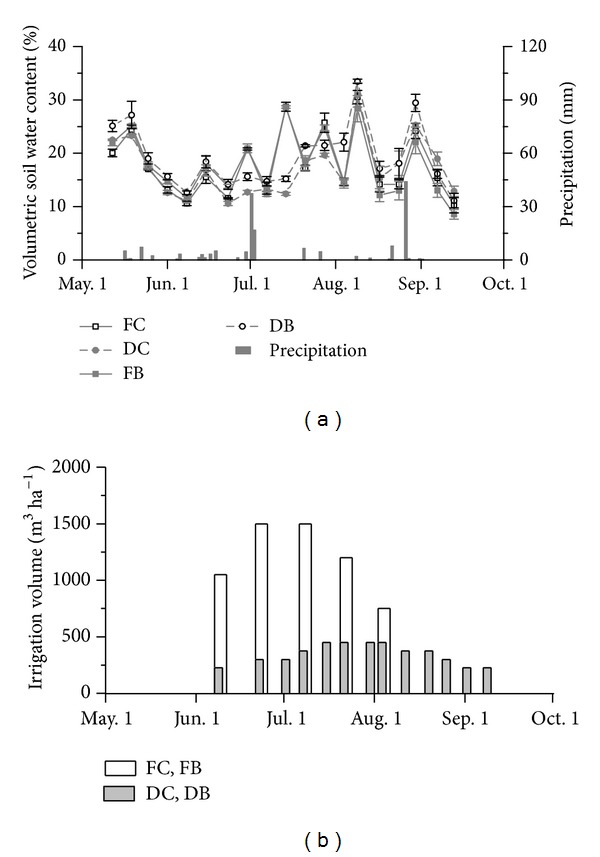
Effects of different irrigation methods and fertilization regimes on soil moisture, precipitation, and volume of irrigation water during cotton growing season.

**Figure 3:**
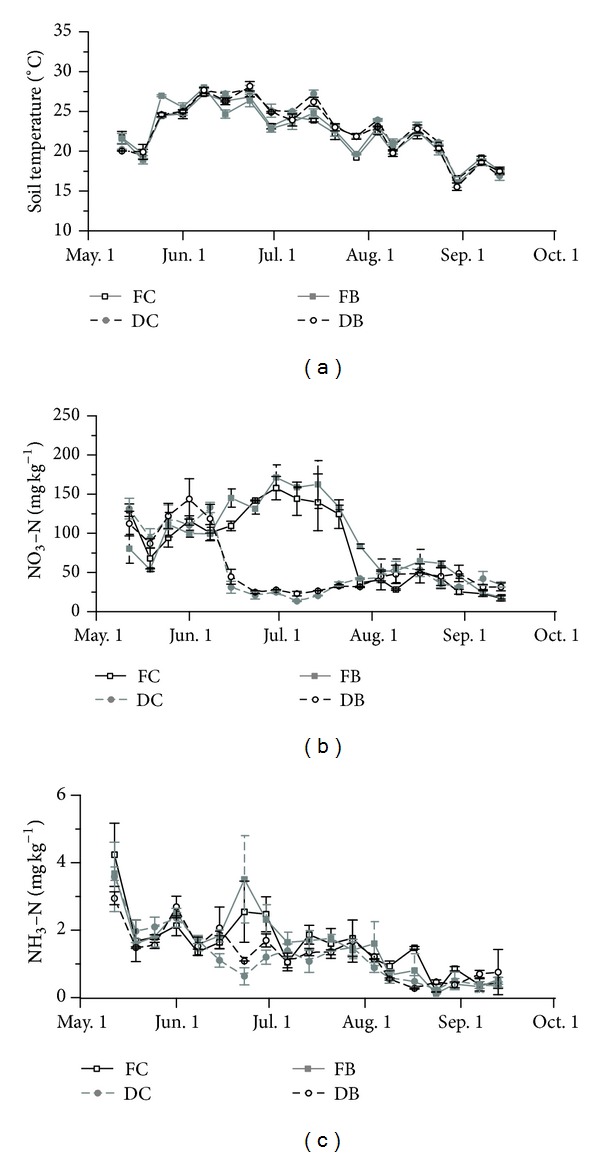
Effects of different irrigation methods and fertilization regimes on soil temperature and mineral N contents during cotton growing season.

The soil moisture under FC and FB was significantly higher than that under DC and DB on June 30, July 14, and July 28, while on other days the soil moisture with FC and FB was close to that with DC and DB (Figure 2(a)). The soil temperatures during cotton growing season were significantly higher with DC and DB than with FC and FB during the bud stage (*P* < 0.05) (Table 2). As compared with FC, DC showed significantly lower soil temperature during flowering and boll-forming stage (*P* < 0.05) (Table 2). However, fertilization regimes had no effect on soil temperature. The soil NO_3_^−^-N contents were significantly higher with FC and FB than with DC and DB during the bud stage, and that with FB was significantly higher than those with FC, DC, and DB during the flowering and boll-forming stage (*P* < 0.05) (Table 2, Figure 3(b)), which was caused by the topdressing of N fertilizer in furrow irrigation plots. This topdressing event also led to a peak in soil NH_4_^+^-N contents with FC and FB on June 23 and June 30 (Figure 3(c)).

Although there was no significant difference between the cotton yield among these four treatments, the water use efficiency (WUE) calculated on cotton yield per unit irrigation volume was significantly higher with DC and DB than that with FC by 53.8% and 60.2%, respectively (*P* < 0.05) (Table 3).

### 3.2. N_2_O Fluxes

**Figure 4:**
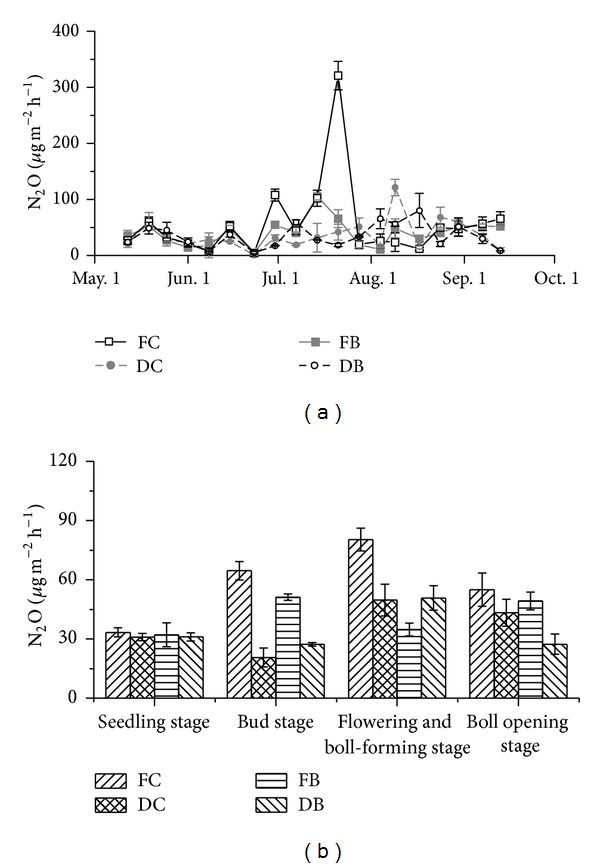
Effects of different irrigation methods and fertilization regimes on N_2_O flux rates during cotton growing season.

The N_2_O flux rates with FC varied from 7.0 *μ*g m^−2^ h^−1^ to 320.9 *μ*g m^−2^ h^−1^ during the cotton growing season, with two flux peaks on June 30 and July 21 (Figure 4(a)). As compared with FC, the N_2_O flux rates of FB were relatively stable and lower. Under DC, the N_2_O flux rates were relatively lower among the sampling dates comparing to FC, except a flux peak on August 9. On most days, the N_2_O flux rates with DB were close to that with DC (Figure 4(a)). The accumulated N_2_O emissions during the cotton growing season were significantly lower with FB, DC, and DB than that with FC by 28.8%, 36.1%, and 37.6%, respectively (*P* < 0.05) (Table 4). At different growth stages, the highest N_2_O flux rate under FC, DC, and DB appeared at the flowering and boll-forming stage, while FB appeared at the bud stage (Figure 4(b)). The N_2_O flux rate with FC performed differently with other three treatments at the flowering and boll-forming stage and the bud stage (*P* < 0.05). No significant difference was observed between DC and DB during the whole period.

### 3.3. CH_4_ Fluxes

**Figure 5:**
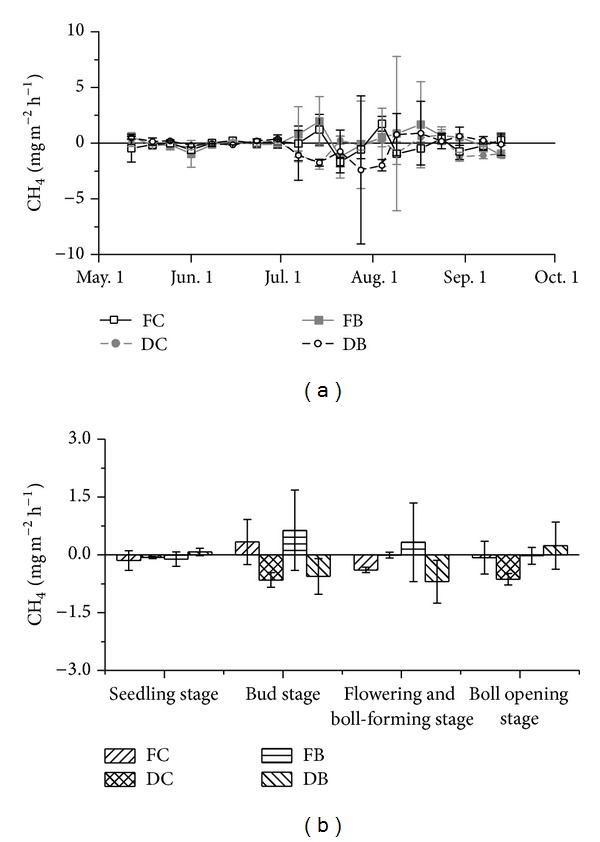
Effects of different irrigation methods and fertilization regimes on CH_4_ flux rates during cotton growing season.

The CH_4_ flux rates under FC, DC, and DB were below zero on most sampling days, indicating that cotton fields were the sink of CH_4_ except under FB for most of the time of the cotton growing season (Figure 5(a)). The CH_4_ flux rates were similar under the four treatments in May and June but diverged after June. The highest uptake of CH_4_ appeared at the flowering and boll-forming stage under FC and DB, while it appeared at the bud stage under DC (Figure 5(b)). The accumulated CH_4_ emissions during the cotton growing season were significantly lower with DC and DB than that with FB by 264.5% and 226.7%, respectively (*P* < 0.05) (Table 4). Although the accumulated CH_4_ emission under FC was 154.2% lower than that with FB, there was little difference between the two treatments.

### 3.4. Yield-Scaled GWP

**Figure 6:**
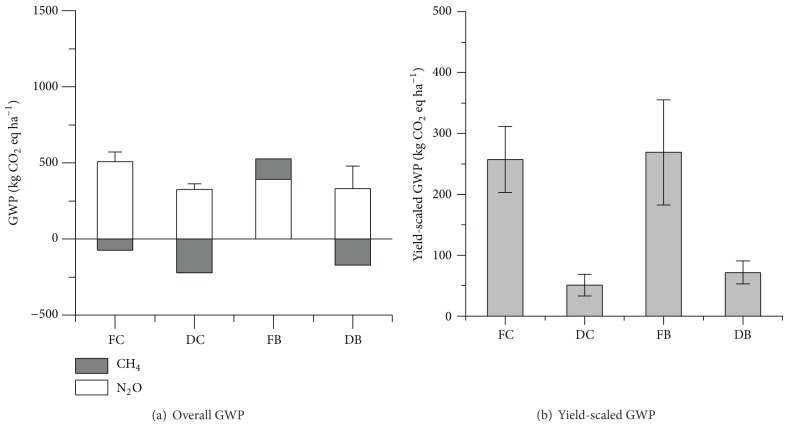
Overall GWP of GHGs (a) and yield-scaled GWP (b) for different treatments. The error bar in (a) was the standard error of overall GWP of N_2_O and CH_4_ emissions.

The total GWP of N_2_O and CH_4_ emissions during cotton growing season was 102.89, 146.23, 435.09, and 496.49 under treatments of DC, DB, FC, and FB, respectively (Figure 6(a)). Cotton fields under DC, DB, and FC were all sinks for CH_4_, which reduced the contribution of N_2_O emission to the overall GWP by 68.3%, 53.9%, and 14.4%, respectively. The yield-scaled GWP calculated by GWP per unit cotton yield with FC and FB were significantly higher than those with DC and DB (*P* < 0.01) (Figure 6(b)). As compared with FC, DC and DB were 80.1% and 72.2% lower in yield-scaled GWP, respectively. Irrigation methods showed extremely significant effect on the total GWP and yield-scaled GWP (*P* < 0.01), while fertilization regimes had no effect on both.

## 4. Discussion

In the present study, it was observed that the soil temperature during cotton growing season was higher with DC and DB than with FC and FB in most of the time during cotton growing season for the plastic film mulching. A similar result was found in the previous study for different irrigation methods in maize field in China [27]. The plastic film usually prevents the evaporation of soil moisture [22]. However, the difference of the soil moisture between four treatments was primarily attributed to the water supply regimes (Figure 2). The plastic film showed little effect on maintenance of soil moisture in the present study. Biochar can efficiently retain soil moisture due to its special physical structure [18], which was consistent with our results under DC and DB.

The emissions of N_2_O and CH_4_ from cotton field were investigated under different irrigation methods and fertilization regimes in an arid area of northwestern China. The accumulated N_2_O emissions during cotton growth season in this study were lower than that from semiarid cotton field in northern China [4] and arid cotton fields in Uzbekistan [6] but higher than the N_2_O emissions from semiarid cotton field in Pakistan [5]. The variance in N_2_O emission among different ecosites might be attributed to the difference in N fertilizer application rate, irrigation, climate factors, and soil properties [28–33]. Here, lower level of N_2_O emission from cotton field was found under FB, DC, and DB, in relation to FC with a high N fertilizer application rate (300 kg N ha^−1^) (Table 4). The relatively lower N_2_O emission from cotton field under drip irrigation treatments was mainly attributed to the water supply regime and N fertilizer dressing method (so-called fertigation) in drip irrigation system, which favored decrease in N_2_O emission. In detail, the soil moisture was lower in cotton field with drip irrigation treatments than that with FC during the bud stage, which was the main fertilization time of FC. Previous studies reported that soil moisture is a key factor in regulation of N_2_O emission from agricultural soil. For instance, the N_2_O emission was enhanced along with increased soil moisture in a given range, due to the improved denitrification [34–36]. Therefore, the relatively lower soil moisture in cotton field with DC and DB during the bud stage could reduce the N_2_O emission more effectively than that with FC. Furthermore, the decreased N fertilizer was directly fertigated to the rhizosphere of cotton plants in drip irrigation treatments with more times but less application rate per time. This N fertilizer dressing method could also improve N uptake of cotton plant but decrease the soil inorganic N pool (NO_3_^−^-N and NH_4_^+^-N) (Table 2) and hence reduce N source for N_2_O emission, which was significantly correlated with soil N [37]. However, the observed reduction in N_2_O emission caused by drip irrigation was less than that in previous studies conducted in the melon and tomato fields [8–10]. This might be related to higher N fertilizer application rate in this study (300 kg N ha^−1^), suggesting that the mitigation effect of drip irrigation on N_2_O may be depressed with a higher N fertilizer application rate.

Although FB and FC used the same irrigation system, FB showed lower N_2_O emission than FC for the addition of biochar. A similar result was found in wheat field [14, 15]. However, the effect of biochar on N_2_O emissions under DB and DC was covered up by the effect of drip irrigation. Biochar had been shown to efficiently retain NH_4_^+^ via cation exchange by its developed specific surface area and surface negative charge density [38]. Then the retained N would be slowly released for plant growth; thus, biochar could coordinate the mineral N availability and plant uptake. This would reduce the amount of N available for denitrification and lost as N_2_O [18]. On the other hand, decreases in emissions of N_2_O in soil amended with biochar might be attributed to improved aeration and porosity for its developed microstructure, which might lead to lower denitrification rates and alter the N_2_O-to-N_2_ ratio during denitrification [16].

The present study indicated that the arid cotton fields under FC, DC, and DB were the sinks of CH_4_, while FB was the source of CH_4_. This was consistent with the results of previous studies with different irrigation methods conducted in upland field [39–41]. However, drip irrigation was a source of CH_4_ in the study in cotton field [22], which was inconsistent with the present results. This might be related to the different rate of irrigation and fertilization. Upland fields are normally net sinks for CH_4_, since the consumption exceeds the production in CH_4_ [42]. The dry soil in upland field limited the CH_4_ emission; however, it did have a possibility to become a source after rainfall within several days or weeks [43, 44]. Comparing the CH_4_ emission between DC and FC, it was shown that DC increased the uptake of CH_4_ for the relative lower soil moisture in most of the time during cotton growing season (Figure 2(a)). The main factor affecting CH_4_ uptake in summer was soil moisture [45], which plays a critical role in CH_4_ consumption [46]. Decrease in soil moisture enhanced CH_4_ oxidation through improving CH_4_ diffusion from atmosphere into soil pore spaces [47] and through gas diffusivity to control microbial oxidation, which changes inversely with soil moisture [48, 49].

In our study, the addition of biochar increased CH_4_ emissions by 284.6% higher with FB than that with FC, while the uptake of CH_4_ with DB was 22.9% lower than that with DC. The result was in good agreement with previous study that biochar addition promoted CH_4_ emissions of the upland soil [17]. However, other studies had reported that biochar amendment reduces CH_4_ emissions as compared with the control [18, 19]. The inconsistence might be attributed to the soil type, agricultural management, and biochar type. On one hand, the addition of biochar increased the substrate supply and created a favorable environment for methanogenic activity. On the other hand, the CH_4_ emission from acid soil was usually much lower than that from neutral soil [50]. Biochar addition could increase soil PH, which was a benefit for methanogenic bacteria. Furthermore, increased soil aeration due to increased porosity could increase CH_4_ diffusion. Biochar might play a more important role under paddy fields, compared with straw returning directly, which would increase CH_4_ emission obviously [51].

There was significant difference between drip irrigation treatments and furrow irrigation treatments in the total GWP of N_2_O and CH_4_ emissions (*P* < 0.01), while fertilization regimes showed little effect (Figure 6). The average yield-scaled GWP was 80.1% and 72.2% lower with DC and DB than that with FC, indicating that drip irrigation could mitigate the yield-scaled GHG emissions from cotton field. In previous studies, cotton yield was higher with drip irrigation than that with furrow irrigation [52], which agreed with our study. Although the difference of cotton yield between four treatments was insignificant, WUE was significantly higher with DC and DB than that with FC by 53.8% and 60.2%, respectively (*P* < 0.05) (Table 3). WUE represented in this study (from 0.263 kg m^−3^ to 0.527 kg m^−3^) were lower than that from the studies in Turkey, where it ranged from 0.508 kg m^−3^ to 0.648 kg m^−3^ or from 0.76 kg m^−3^ to 1.46 kg m^−3^ [52, 53]. The variance in WUE in different ecosites could be related to climate, plant number, varietal differences, and irrigation amount [54]. It indicated that drip irrigation significantly increased WUE of cotton plants, in relation to furrow irrigation. The relative higher WUE with DC and DB was related to improved fertilizer efficiency and depressed leaching potential in drip irrigation system [55].

## 5. Conclusions

Irrigation methods significantly affected the GHG emissions from cotton field in arid northwestern China. Biochar addition increased CH_4_ emissions and decreased N_2_O emissions. The water supply and N fertilizer dressing method played a key role in regulating gas emissions. Drip irrigation treatments (DC and DB) remarkably reduced GHG emissions, compared with furrow irrigation treatments (FC and FB). In addition, drip irrigation treatments (DC and DB) had higher yield and WUE, compared to furrow irrigation treatments (FC and FB). Thus, mulched-drip irrigation with conventional fertilization or conventional fertilization + biochar should be a better approach to increase cotton yield with depressed GHG emissions in arid northwestern China.

## Figures and Tables

**Table 1:** The applications of irrigation and topdressing during the cotton growth period.

Irrigation date	Drip irrigation treatments			Furrow irrigation treatments		
	Volume (m^3^ *·*hm^−2^)	Urea (kg*·*hm^−2^)	Potassium dihydrogen phosphate (kg*·*hm^−2^)	Volume (m^3^ *·*hm^−2^)	Urea (kg*·*hm^−2^)	Potassium dihydrogen phosphate (kg*·*hm^−2^)
6.10-6.11	225	30	15	1050	150	
6.24-6.25	300	30	15	1500	300	60
7.3	300	30				
7.9-7.10	375	60		1500	105	30
7.17	450	75				
7.23-7.24	450	75		1200		
8.1	450	60				
8.5-8.6	450	60		750		
8.13	375	45	15			
8.21	375	45	15			
8.27	300	30	15			
9.3	225	15	15			
9.10	225					

**Table 2:** Effects of different irrigation methods and fertilization regimes on major soil characteristics. Values are means ± standard deviation of three replicates. Different small letters in the same column refer to significant difference between treatments at *P* < 0.05 level.

	Seedling stage	Bud stage	Flowering and boll-forming stage	Boll opening stage
Soil moisture (%)				
FC	16.79 ± 0.37b	18.63 ± 0.25a	20.46 ± 1.04a	16.00 ± 1.25b
DC	17.28 ± 0.05b	12.24 ± 0.13c	19.79 ± 1.00a	18.81 ± 1.13a
FB	18.19 ± 0.35ab	18.92 ± 0.35a	19.52 ± 0.85a	14.16 ± 1.50b
DB	19.65 ± 0.71a	14.93 ± 0.69b	23.11 ± 0.82a	18.70 ± 1.56a
Soil temperature (°C)				
FC	24.01 ± 0.33a	24.55 ± 0.12b	21.2 ± 0.12b	18.53 ± 0.10a
DC	23.98 ± 0.12a	26.29 ± 0.25a	22.55 ± 0.15a	18.29 ± 0.27a
FB	24.28 ± 0.19a	24.41 ± 0.36b	21.78 ± 0.37ab	18.03 ± 0.37a
DB	23.92 ± 0.09a	25.80 ± 0.37a	22.11 ± 0.33ab	18.00 ± 0.05a
Soil NO_3_^−^-N (mg kg^−1^)				
FC	100.82 ± 4.03a	145.82 ± 3.52a	56.38 ± 6.68b	26.94 ± 4.18a
DC	108.44 ± 3.39a	19.80 ± 1.44b	46.11 ± 1.29b	38.77 ± 5.15a
FB	98.24 ± 6.86a	155.94 ± 12.38a	76.94 ± 2.49a	37.26 ± 0.12a
DB	104.75 ± 5.31a	25.54 ± 0.80b	41.13 ± 4.90b	39.19 ± 6.45a
Soil NO_4_^+^-N (mg kg^−1^)				
FC	2.14 ± 0.28a	1.98 ± 0.41ab	1.38 ± 0.19a	0.46 ± 0.08a
DC	2.14 ± 0.21a	1.07 ± 0.13b	0.96 ± 0.01a	0.36 ± 0.08a
FB	2.17 ± 0.05a	2.29 ± 0.16a	1.24 ± 0.24a	0.34 ± 0.05a
DB	2.04 ± 0.11a	1.29 ± 0.06b	1.01 ± 0.12a	0.57 ± 0.16a

**Table 3:** Effects of different irrigation methods and fertilization regimes on cotton yield and water use efficiency. Values are means ± standard deviation of three replicates. Different small letters in the same column refer to significant difference between treatments at *P* < 0.05 level.

	FC	DC	FB	DB
Cotton yield (Mg ha^−1^)	1.76 ± 0.16a	2.02 ± 0.10a	1.94 ± 0.17a	2.11 ± 0.14a
Water use efficiency (kg m^−3^)	0.29 ± 0.03b	0.45 ± 0.02a	0.32 ± 0.03b	0.47 ± 0.03a

**Table 4:** Effects of different irrigation methods and fertilization regimes on accumulated N_2_O and CH_4_ emissions during cotton growing season. Values are means ± standard deviation of three replicates. Different small letters in the same column refer to significant difference between treatments at *P* < 0.05 level.

	N_2_O (kg ha^−1^)	CH_4 _(kg ha^−1^)
FC	1.71 ± 0.13a	−2.92 ± 0.96ab
DC	1.09 ± 0.11b	−8.87 ± 1.85b
FB	1.21 ± 0.07b	5.39 ± 4.91a
DB	1.04 ± 0.06b	−6.84 ± 1.07b

## Abbreviations

GHG: Greenhouse gas
GWP: Global warming potential
WUE: Water use efficiency.
